# Intermittent fasting and neuroprotection in Alzheimer’s disease: metabolic mechanisms, cellular signaling, and brain-peripheral crosstalk

**DOI:** 10.3389/fnut.2026.1839995

**Published:** 2026-05-25

**Authors:** Keshari H. Sudasinghe, Stephanie E. Hall

**Affiliations:** Department of Anatomy and Physiology, College of Veterinary Medicine, Kansas State University, Manhattan, KS, United States

**Keywords:** adipose tissue, Alzheimer’s disease, brain health, brain metabolism, intermittent fasting, skeletal muscle

## Abstract

Intermittent fasting (IF) promotes a metabolic switch characterized by reduced glucose and insulin availability along with increased lipolysis and ketone body production, particularly *β*-hydroxybutyrate (βOHB). In the brain, IF enhances metabolic flexibility by facilitating ketone utilization and supporting the astrocyte–neuron lactate shuttle (ANLS), partially compensating for cerebral glucose hypometabolism which is commonly observed in Alzheimer’s disease (AD). Beyond bioenergetics, IF activates autophagy and inhibits mTOR signaling, promoting protein clearance and cellular homeostasis. Neuroinflammation is also attenuated with IF through the modulation of microglial activation. IF further induces increased levels of brain-derived neurotrophic factor (BDNF), thereby supporting synaptic plasticity and neuronal resilience. At the systemic level, IF enhances brain-peripheral crosstalk by improving adipose tissue function (e.g., leptin sensitivity and adipokine balance) and stimulating skeletal muscle–derived myokine signaling, which collectively influence brain metabolism and inflammation. These integrated mechanisms converge to reduce amyloid-β accumulation, tau pathology, and neuroinflammation, ultimately improving synaptic function and cognitive outcomes, as evidenced by preclinical rodent models and emerging clinical studies.

## Graphical abstract

**Figure:**
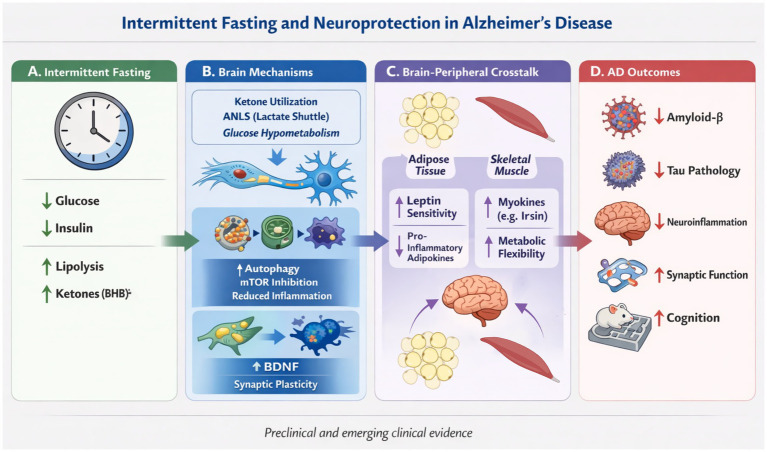

## 1 Introduction

Intermittent fasting (IF) is a dietary regimen that incorporates periods of fasting with or without overall calorie restriction. This regimen began as a trendy weight-loss strategy but has since gained significant research interest for its broad health benefits. IF has been shown to promote health and reduce the risk of various diseases, including cardiovascular disease, obesity, and type 2 diabetes (1–3). Beyond these metabolic benefits, clinical research suggests that IF may also support aspects of brain health, including improvements in neurodegenerative disease pathologies (4). There are several popular methods of IF, including (1) the 16/8 regimen (16 h of fasting followed by an 8-h eating window) (5), (2) the ADF, (alternate day fasting, alternating between fasting days with zero or extremely low caloric intake and non-restricted feeding days) (6), and (3) the 5:2 IF regimen (2 days per week of reduced energy intake and normal intake on the remaining 5 days) (7). While these methods differ in implementation, the key point is that they do not dictate where the calories come from, i.e., low-fat or low-carbohydrate; the most important part is the incorporation of a fasting period.

This review will examine contemporary evidence from human and rodent studies examining the impact of IF on brain health, brain aging, and neurodegenerative pathology with a particular focus on Alzheimer’s disease (AD). Specifically, it will begin with an understanding of brain metabolism during fasting, when the brain relies less on glucose and more on ketones, followed by a discussion of metabolic impairment in AD. The second section of this review will focus on mechanisms of IF-induced neuroprotection, including neuroinflammation and autophagy. Lastly, the review will explore the role of peripheral-central signaling, particularly how adipose tissue and skeletal muscle communicate with the brain to enhance neuronal function and resilience against neurodegenerative pathology, with a specific emphasis on AD.

## 2 Metabolic mechanisms of IF-induced neuroprotection

The brain is a significant energy consumer; although it accounts for only 2% of body weight, it uses approximately 20% of the body’s total energy. This energy is primarily from the metabolism of blood glucose through glycolysis and the tricarboxylic acid (TCA) cycle. Glucose enters the brain via glucose transporters (GLUT) located at the blood–brain barrier (BBB). Most glucose is transported into astrocytes via GLUT1, where it is metabolized into lactate before being transported to neurons via the lactate shuttle (8). Neurons can also directly take up glucose via GLUT3, although this is less common. In states of altered metabolism, such as those associated with neurodegeneration, these processes are disrupted (9), as discussed in subsequent sections.

### 2.1 Ketone utilization as an alternative brain fuel

Ketone bodies are produced in the liver through the oxidation of fatty acids. In the brain, they become an important alternative fuel source during periods of low glucose availability, such as fasting, exercise, or carbohydrate restriction, by entering mitochondrial oxidative phosphorylation to generate ATP. IF promotes the production of ketones such as β-hydroxybutyrate (βOHB) and acetoacetate, which serve as alternative energy substrates when glucose metabolism is impaired (10). When glucose availability is low, fatty acids are broken down into acetyl-CoA, which accumulates and is converted to acetoacetate in the hepatic mitochondria, where it can be reduced to βOHB. Both of these ketone bodies circulate in the bloodstream and serve as alternative energy substrates for the brain (11).

Neurodegenerative diseases are characterized by reduced cerebral energy availability due to impaired glucose uptake (reduced expression of GLUT1 and GLUT3) and utilization (increased brain insulin resistance), as well as mitochondrial dysfunction (limiting ATP production), all of which contribute to cognitive and neuropathological impairments (12–14). Notably, preclinical studies have demonstrated that 20 weeks of ADF increases circulating βOHB levels and enhances brain ketone metabolism in 3xTg-AD mice, while also improving cognitive functions (10). ADF intervention also reduced AD pathology, specifically decreasing amyloid beta oligomers and tau hyperphosphorylation in the hippocampus (10). During prolonged fasting (three obese patients undergoing 38–41 days of fasting), ketones, such as βOHB, can provide approximately 60–70% of the total energy required for brain function (15, 16). This metabolic shift is facilitated by increased expression of the ketone transporter, monocarboxylate transporter 1 (MCT1), at the blood–brain barrier, thereby enhancing the brain’s capacity to utilize ketones (17).

### 2.2 Ketones as signaling molecules

In addition to serving as an alternative energy substrate, ketone bodies cross the BBB and function as a potent signaling molecule in the brain (18). *In vitro* studies show that ketones such as βOHB induce brain-derived neurotrophic factor (BDNF) expression in cerebral cortical neurons (19). BDNF is a key regulator of brain plasticity and neurogenesis, thereby linking ketone signaling to neuroprotection and cognitive resilience (20). In addition, βOHB can inhibit class 1 histone deacetylases (HDACs) and promote histone beta-hydroxybutyrylation, leading to the activation of gene expression linked to oxidative stress resistance and neurotrophic signaling (21). Furthermore, βOHB can also bind to free fatty acid receptor-3 (FFAR3), a receptor expressed in sympathetic ganglia, leading to suppression of sympathetic activity and reduction in metabolic rate in mice (22).

### 2.3 Lactate and the astrocyte-neuron lactate shuttle (ANLS)

Blood lactate serves as an important energy substrate for the human brain. In fact, up to 20% of cerebral energy is derived from lactate under eustress, such as during exercise or fasting (23). The brain’s reliance on lactate as a fuel source has also been confirmed in humans following 3 days of fasting (24). A study by Pan et al. (24) demonstrated that human brain lactate levels rise after fasting, reporting that non-fasted brain lactate was 0.69 ± 0.17 mmol/L, which increased to 1.47 ± 0.22 mmol/L after 3 days of fasting. This indicates a critical role in maintaining brain energetics during fasting.

Within the brain, lactate serves as an energy substrate via the Astrocyte-Neuron Lactate Shuttle (ANLS). Astrocytes take up glucose from the bloodstream and metabolize it to pyruvate via glycolysis. Pyruvate is then converted to lactate by the enzyme lactate dehydrogenase (LDHA). Lactate is exported from astrocytes into the extracellular space via monocarboxylate transporters, MCT1 and MCT4, which are expressed in the astrocytes and adapted for lactate export within the brain. Neurons then take up lactate via MCT2, which has a high affinity for lactate. Once inside the neuron, lactate is converted back into pyruvate and oxidized in the mitochondria to produce energy (25). Disruption of MCTs impairs lactate transport, leading to neuronal energy deficits within the brain (26).

In addition to serving as an energy source, lactate promotes brain plasticity by modulating N-methyl-D-aspartate receptor (NMDAR) signaling. NMDARs are glutamate receptors and play key roles in synaptic plasticity and synaptogenesis. Lactate enhances neuronal signaling by increasing calcium influx and inward current following glutamate activation of the NMDA receptor (25). In that context, lactate acts as a signaling molecule that strengthens NMDARs. A study by Yang et al. (27) shows that lactate induces the expression of plasticity genes such as Arc, c-Fos, and Zif268 in neurons by potentiating NMDA signaling and downstream ERK1/2 signaling.

### 2.4 Metabolic impairments in AD

Glucose transporters such as GLUT 1 (expressed primarily on endothelial cells at the brain barrier), GLUT3 (expressed primarily in neurons throughout the brain), and GLUT4 (expressed in hippocampal neurons throughout the brain) are essential for cerebral glucose uptake. Clinical studies show that deficiencies in GLUT1 and GLUT3 impair glucose metabolism and may contribute to neurodegenerative pathology. For example, a study reported that in postmortem AD cortex, both GLUT1 and GLUT3 levels were decreased by 25–30% compared with controls, and these reductions correlated with increased tau hyperphosphorylation and neurofibrillary tangle density (12).

Preclinical studies in mice overexpressing human mutated Alzheimer amyloid β (Aβ) precursor protein (APP) show that GLUT1 deficiency leads to early BBB breakdown, cerebral microvascular degeneration, and accelerated neuronal pathologies (28). In AD, GLUT1 reduction may result from endothelial cell damage, which can reduce GLUT1 expression at the BBB, or from amyloid-beta toxicity (28). Glucose hypometabolism in AD occurs early and is driven largely by reduced expression of GLUT1/3 rather than by neuronal loss, thereby limiting neuronal glucose uptake and compromising energy production. In triple transgenic AD mice (3XTg-AD mice, which express human gene mutations in APP, PSEN1, and MAPT), glucose hypometabolism preceded neuronal death and is associated with increased Aβ and tau phosphorylation, suggesting that metabolic impairments exacerbate Aβ and tau pathology (29), characterized by the accumulation of Aβ into extracellular plaques (14) and intracellular aggregation of hyperphosphorylated tau into neurofibrillary tangles (30).

Beyond impaired glucose transport, disruption in brain insulin signaling also contributes to metabolic dysfunctions in AD. Unlike peripheral tissues, the majority of glucose uptake in the brain is insulin-independent and relies largely on GLUT1/GLUT3 glucose transporters; however, Insulin signaling in the brain remains critical for neuronal survival and energy homeostasis. A study by Schubert et al. (31) shows that insulin receptors are required to inhibit apoptosis in neuronal cells. Administration of insulin directly into the brain inhibits food intake and body weight, whereas deletion of neuronal insulin receptors leads to increased food intake (hyperphagic) and obesity (32, 33). In addition, impaired neuronal insulin signaling has been linked to neurodegenerative pathology, as neuron-specific insulin receptor knockout mice exhibit increased tau hyperphosphorylation (31).

Clinical evidence shows that brain glucose uptake is impaired in patients with AD and mild cognitive impairment (MCI), whereas brain ketone uptake remains normal in both MCI and early AD (34). Under a normal mixed diet, plasma ketone levels are usually low (<0.5 mM), accounting for less than 5% of brain energy metabolism (11). During a ketogenic diet (KD), plasma ketone concentrations can increase 8-fold. After 4 days of KD, the cerebral metabolic rate of ketones can reach about 17% of the whole brain energy requirement in healthy adults (13), suggesting that diet-induced ketones can serve as an alternative fuel to glucose for the human brain. In AD, glucose metabolism is impaired, possibly due to dysfunction of glucose transporters as previously described, and aging is associated with a marked reduction in cerebral glucose metabolism (35, 36). This has been linked to cognitive decline, suggesting that ketone as an alternative fuel may be beneficial in age-related neurodegenerative diseases.

## 3 Non-metabolic mechanisms of IF-induced neuroprotection

### 3.1 IF reduces neuroinflammation and modulates microglia

Microglia are the innate immune cells of the central nervous system (CNS) and play a protective role by clearing debris via phagocytosis. This cellular process involves the engulfment and removal of damaged cells and cell debris (37). Specifically, microglia are also critical for the clearance of Aβ in the brain. The receptor Triggering Receptor Expressed on Myeloid cells 2 (TREM2) facilitates the clearance of aggregated proteins, bacteria, and apoptotic neurons, and low-density lipoproteins in the brain. Apolipoproteins such as ApoE/ApoJ activate the TREM2 and signal through its adaptor protein DAP12, which in turn activates downstream effectors, including phosphatidylinositol 3-kinase (PI3K) and Ca2+ signaling pathways, which promote microglial phagocytosis (38). In AD, however, microglia become proliferative and morphologically altered, forming clusters around the Aβ plaques, but their containment capacity becomes reduced and overwhelmed as plaque deposition increases. When microglial containment fails, Aβ fibrils serve as a scaffold for further Aβ aggregation, generating plaques that spread into nearby neurons and exacerbate pathology. These reactive microglia also release inflammatory cytokines such as interleukin-1β (IL-1β) and tumor necrosis factor-α (TNF-α), which promote synaptic loss and exacerbate tau pathology (38). For example, overexpression of IL-1β increases tau phosphorylation and reduces synaptic protein levels in neurons via activation of neuronal p38 mitogen-activated protein kinase (p38-MAPK) phosphorylation. p-38 MAPK is significantly increased in neurons treated with recombinant IL-1β within the microglia, and the inhibition of p38-MAPK significantly suppresses IL-1β- induced tau phosphorylation (39).

Disruption of intercellular lipid metabolism and transport capacity, leading to pathological accumulation of lipid droplets within microglia (40). Studies have shown that lipid-accumulating microglia, characterized by prominent lipid droplets, exhibit impaired phagocytosis and TREM2 signaling (40). Importantly, IF significantly decreased the accumulation of lipid droplets within microglia, and these microglia with fewer lipid droplets may be involved in more phagocytic activity, thereby reducing Aβ deposition in the mouse brain with IF intervention (41). Similarly, Wu et al. (41) demonstrated that six-month-old mice subjected to 1 month of IF exhibited improved cognitive function, along with microglial morphological changes characterized by enlarged cell bodies (soma), reduced process complexity, and fewer lipid droplet accumulations, adaptations that enhanced their phagocytic capacity and promoted engulfment and clearance of Aβ.

Brain inflammation occurs when immune cells respond to infection or other harmful stimuli, and it is primarily mediated by microglia and astrocytes (42). While acute inflammation can be protective, chronic neuroinflammation disrupts neuronal function and drives neurodegenerative diseases such as AD. Studies have found that stressed or apoptotic adipocytes recruit macrophages and secrete inflammatory mediators, including lipocalin 2 (LCN2) and galectin 3 (GAL3) (43). These mediators cross the BBB and bind neuronal receptors, activating astrocytic JAK/STAT3 and microglial TLR4 pathways, thereby driving downstream inflammation. These adipose-derived inflammatory mediators, driving astrogliosis (which is proliferation and activation of reactive astrocytes characterized by enlarged soma morphology) (44) and microgliosis (which is known as activated microglia and proliferative microglia within the CNS), lead to the release and secretion of cytokines that impair neuronal functions and synaptic plasticity-induced astrocytic LCN2 and microglial GAL3 protein expression in the brain hippocampus (43).

### 3.2 IF activates autophagy and inhibits mTOR signaling

Autophagy is a catabolic process that degrades and recycles cytosolic components through the lysosomal-derived proteolytic pathway (45). This process involved several key stages: initiation [activation of Unc-51-like autophagy activating kinase 1 (ULK-1)], nucleation (activation of beclin-1), elongation [light chain3-II (LC3II)/autophagy protein-5 (Atg5)], fusion (with the lysosome), and final degradation via lysosomal proteolysis (46). In the healthy brain, autophagy mediates the degradation of proteins and organelles during nutrient starvation, whereas nutrient sufficiency suppresses excessive activation and maintains autophagy flux (47, 48). Suppression of basal autophagy causes neurodegeneration by impairing autophagic flux, leading to toxic protein accumulation and neuronal loss (49). For instance, mice lacking autophagy-related protein 5 (Atg5), a protein required for autophagosome membrane elongation, developed progressive motor and behavioral defects after 3 weeks of age. They developed neurodegenerative changes, such as axon swelling, in the brain, specifically in Purkinje cells, the cerebral cortex, the hippocampus, and the cerebellum, by 3 months of age. Aging is also associated with a progressive decline in autophagy, and inducing cellular autophagy can reverse aging phenotypes. For example, systemic overexpression of Atg5 in mice has been shown to extend lifespan by 17% (50).

Studies further revealed that mammalian target of rapamycin (mTOR), an upstream regulator of autophagy, is critical in regulating autophagy and provides protection against aging and neurodegenerative diseases (51). mTOR plays a central role in regulating energy metabolism, and activation of the mTOR pathway inversely correlates with autophagy (47). mTOR functions in two distinct complexes, mTORC1 and mTORC2. mTORC1 (mammalian target of rapamycin complex 1) integrates nutrient, energy, and growth factor signals to promote anabolic processes while suppressing catabolic processes, whereas mTORC2 (mammalian target of rapamycin complex 2) regulates cell survival and cytoskeletal organization (51). mTORC1 is active in nutrient-rich environments (fed stage), during which autophagy remains at a basal level. In contrast, nutrient deprivation decreases mTORC1 activity and upregulates autophagy (52).

In AD, hyperactivation of mTOR signaling has consistently been observed (52, 53). Babygirija et al. (54) demonstrated that in 3xTg-AD mice, calorie restriction (CR) combined with fasting reduced phosphorylation of mTOR substrates, eukaryotic translation initiation Factor 4E Binding protein 1 (P-4EBP1), a downstream regulator of mTOR. Reduction of 4EBP1 phosphorylation prevents initiation of protein translation as unphosphorylated 4EBP1 binds tightly to eIF4E (a cap binding protein), thereby blocking translation.

Cellular energy balance is frequently monitored by adenosine monophosphate-activated protein kinase (AMPK), an upstream regulator of mTOR. AMPK closes pathways involved in energy consumption, such as mTOR activation. AMPK activates a protein known as the Tuberous Sclerosis Complex (TSC), and loss of TSC1/TSC2 inactivates the TSC protein complex and leads to hyperactivation of mTOR (55, 56). Hyperactivation of mTOR (Losing the inhibitory effect of mTOR) leads to the inactivation of proteins responsible for autophagy, such as ULK1 (initiates autophagy), light chain 1 (LC3I, elongation of the autophagosome), and light chain II (LC3II, maturation of the autophagosome) and microtubule-associated protein A/1B-light chain 3 (LC3) (57). Typically, activation of AMPK, a sensor of low cellular energy, leads to inhibition of mTORC1, thereby reducing anabolic processes while promoting catabolic processes (57). However, AD brains exhibit concurrent activation of both AMPK and mTOR in neurons with tau pathology and oxidative damage (53). This coactivation suggests a disruption in the usual inhibitory link between AMPK and mTOR, thus allowing mTOR to remain hyperactive despite AMPK signaling and potentially contributing to impaired catabolic processes. Furthermore, our own work in Fischer-344 rats exposed to ADF demonstrated that cortical mTOR reduction occurred independently of TSC (90), suggesting an alternative regulatory mechanism. For example, AMPK can directly inhibit mTORC1 by phosphorylating the mTOR binding partner raptor on Ser 722/792 (58).

IF shares several key metabolic and cellular features with other lifestyle-based interventions, including KD, CR, and exercise, but is distinguished by the timing of these responses (Table 1). Specifically, IF promotes intermittent increases in circulating ketone bodies during fasting cycles, whereas KD induces a more sustained state of ketosis, and exercise typically produces transient elevations that depend on intensity and duration (15, 24). In parallel, IF and CR both activate autophagy through AMPK activation and mTOR inhibition; however, CR has shown a more robust autophagic response. Exercise also initiates autophagy but to a more variable extent, largely dependent on exercise intensity (45, 47, 51).

**Table 1:** A qualitative comparison of IF, KD, CR and exercise.

Intervention	Ketone response	Autophagy	AD-related outcomes
Intermittent fasting (IF)	Intermittent elevations in ketone bodies during fasting (15, 24)	Autophagy activation via AMPK–mTOR signaling (45, 47)	Reduced amyloid-β burden and improved cognition in AD models (10, 59)
Ketogenic diet (KD)	Sustained ketosis due to continuous carbohydrate restriction (11, 13)	Autophagy activation via mTOR and Sirt1 (93, 94)	Mixed-to-positive cognitive and metabolic outcomes in AD contexts (11, 34)
Caloric restriction (CR)	Moderate increases in ketone utilization (15, 34)	Strong and consistent activation of autophagy (47, 51)	Robust improvements in AD-related pathology and behavior in models (54, 59)
Exercise	Transient increases in ketone bodies depending on intensity and duration (5, 73)	Variable induction of autophagy and mitophagy (73, 76)	Generally positive effects on brain function and resilience (72, 74)

This table is intended as a qualitative summary rather than a systematic comparison.

All these interventions have been associated with neuroprotective effects in AD, including reductions in amyloid-β pathology and improvements in cognition. However, outcomes vary by intervention, with CR and IF showing more consistent benefits in preclinical models, whereas the effects of KD and exercise tend to be more variable (10, 54, 59). Taken together, IF can be viewed as a temporally distinct metabolic strategy that intermittently engages many of the same protective pathways activated by these related interventions, while potentially offering unique advantages through repeated metabolic switching (1).

## 4 Brain crosstalk mechanisms of IF-induced neuroprotection

### 4.1 Brain-adipose tissue crosstalk: IF improves leptin sensitivity and adipokine balance

Leptin is a hormone-adipokine secreted by adipose tissue that plays a crucial role in regulating hunger, body weight, and metabolism (60). A study by Annweiler et al. (61) suggests that a non-linear U-shaped relationship exists between circulating leptin levels and cognitive performance in older individuals, indicating that both lower and higher leptin concentrations are associated with impaired cognition. Importantly, leptin crosses the BBB and influences hippocampal synaptic transmission by regulating NMDA receptors, a class of glutamate receptors that mediate excitatory neurotransmission in several brain regions. Leptin enhances NMDA receptor activation and promotes hippocampal long-term potentiation (LTP), a long-lasting enhancement of synaptic communication (62). Indeed, animals with impaired leptin receptor signaling show disrupted hippocampal plasticity characterized by weakened LTP (63).

Leptin also has a dual role in inflammation; in obese individuals, leptin acts as a pro-inflammatory mediator. A study by Poosri et al. (64) showed that individuals with obesity had significantly higher leptin levels, which correlated positively with TNF-alpha and IL-6, both pro-inflammatory mediators. Leptin has been shown to activate immune cells to secrete pro-inflammatory mediators via activation of Janus kinase 2/activator of transcription 3 (JAK2/STAT3) and mitogen-activated protein kinase/extracellular signal-regulated kinase (MAPK/ERK) signaling pathways (65). In addition, Poosri et al. (64) showed that high total fat intake increased leptin levels, contributing to diet-driven hyperleptinemia. Beyond its pro-inflammatory role, leptin can also exert anti-inflammatory effects, such as stimulating innate immunity by activating natural killer cells, which play a key role in identifying and eliminating virus-infected cells. Lo et al. showed that leptin signaling is critical for natural killer (NK) cell development, as leptin-deficient mice exhibit reduced bone marrow NK cell numbers due to increased apoptosis (66). In addition, recombinant leptin can rescue NK cell survival *in vitro*. Supporting this, Tian et al. (67) reported that impaired leptin signaling impaired NK cell activation, further implicating leptin as a key regulator of NK cell function. High-fat diet (HFD) fed mice also showed that 10 days of HFD feeding causes NK cell dysfunction and increased susceptibility to viral infection (68), suggesting that diet may affect NK cell regulation and that leptin likely plays a crucial role in immune regulation.

Several studies have shown that IF improves leptin sensitivity in preclinical models (69). A 16 h fast and an 8-h feeding regimen improved leptin sensitivity in obese mice, along with metabolic benefits, such as improved glucose metabolism, thermogenesis, and reduced fat mass; these metabolic benefits were absent in leptin-deficient mice (69). Conversely, mice fed a high-fat diet and subjected to IF (24 h of feeding and 24 h of fasting for 4 weeks) exhibited decreased leptin levels and reduced leptin gene expression (in both hypothalamus and epididymal fat), while improving adiponectin levels, another adipose-derived adipokine that is strongly linked to anti-inflammation (70, 71).

### 4.2 Brain-skeletal muscle crosstalk: IF promotes skeletal muscle metabolic adaptations and myokine signaling

Lifestyle interventions such as exercise promote the release of myokines, including BDNF, into the systemic circulation, where they act on the brain to improve brain health and synaptic plasticity. Notably, a study by Yang et al. (72) provides evidence that muscle-specific BDNF plays an important role in energy metabolism during fasting, as BDNF increases mitochondrial respiration and lipid oxidation in muscles. In addition, 24-h fasting elevated BDNF expression in gastrocnemius, extensor digitorum longus (EDL) and tibialis anterior in female mice; however, no changes in BDNF expression were observed in fasted males, suggesting that muscle-derived BDNF expression in response to fasting may be sex-specific (72). Interestingly, in BDNF-knockout mice, nutrient stress impaired energy metabolism while promoting muscle weakness, as evidenced by reduced O_2_ consumption, lower energy expenditure, impaired autophagy, a higher respiratory exchange ratio (RER), and insulin resistance (72).

The paradigm that reducing calorie intake improves mitochondrial health is widely accepted; for example, under dietary restriction, AMPK upregulates peroxisome proliferator-activated receptor gamma coactivator alpha (PGC-1α) in skeletal muscle (73). PGC-1α is a master regulator of mitochondrial biogenesis and functions. Because mitochondrial impairment is a hallmark of AD (74), this pathway may be particularly relevant for preserving metabolic health and muscle mass with IF (75). Previous studies have shown skeletal muscle loss during fasting, yet AMPK appears to be essential for metabolic adaptations (76). In AMPK knockout mice, prolonged fasting led to hyperglycemia and hyperketosis, along with impaired autophagy markers, including ULK1. This study indicates AMPK is required to maintain blood glucose during prolonged fasting, which may be critical for glucose signaling in the brain (76). PGC-1α has also been shown to prevent activation of the catabolic system and protect against disuse muscle atrophy (77). Together, these findings suggest that fasting-induced AMPK activation in skeletal muscle exerts protective effects primarily through the AMPK-PGC-1α cascade (77).

Interestingly, Liu et al. (78) reported that a 10-week IF regimen significantly improved locomotion, motor coordination, and muscular strength compared with controls. Immunohistochemistry revealed increased expression of myelin-related proteins within axons, suggesting enhanced myelin integrity and repair. These findings indicate that IF not only preserves muscle functions but also supports neuromuscular health, ultimately mitigating age-related motor and cognitive decline (78).

### 4.3 Evidence of IF’s beneficial effects in AD from preclinical models

Transgenic preclinical models (APP/PS1, 3xTg) provide strong evidence that fasting mitigates AD pathology (Table 2). In rodent models, fasting has been shown to reduce amyloid accumulation (35, 54, 79) diminish tau pathology (10, 54), and lower brain inflammation (41, 54). A recent study in 3xTG-AD mice demonstrated that while both CR without fasting and with fasting reduce amyloid plaque burden in females, only CR with fasting improved cognitive function, reduced neuroinflammation, and reduced both amyloid plaque burden and tau pathology (54).

**Table 2:** Preclinical and clinical outcomes of IF.

Study type	Model/population	IF protocol	Duration	Measured outcomes	Key findings	Reference
Pre-clinical	APP/PS1 mice	IF	30 days	Cognition Aβ Microglial lipid droplets (LDs)	↑ cognition ↓ Aβ ↓ LDs	(41)
Pre-clinical	3xTg-AD young mice	CR + Fasting	9 months	Cognition BDNF Insulin sensitivity (Insulin S) Aβ, tau	↑ cognition, BDNF, and Insulin S ↓ Aβ and tau	(54)
Pre-clinical	3xTgAD mice male	ADF	20 weeks	βOHB Cognition Anxiety	↑βOHB ↓anxiety	(10)
Pre-clinical	3xtg-AD rats young	IF	7 months/14 months	Memory Aβ, tau	Attenuate cognitive deficits (14 m) no changes in Aβ, Tau	(59)
Clinical	Obese older adults	IF	8 weeks	Memory CSF Aβ/tau	↑memory No change in CSF Aβ/tau	(4)
Clinical	Adults	IF	26 weeks (1–2, 24 h fasts per week)	Metabolic syndrome score (MSS) Cognition Insulin Resistance (IR) BDNF	↓MSS score ↓IR No changes in memory and BDNF	(87)
Clinical	Healthy adults	Ramadan fasting	3 weeks	IL-1β, IL-6, TNF-α Body fat Blood pressure (BP)	↓ IL-1β, IL-6, and TNF-α ↓ body fat and BP	(88)
Clinical	Adults with MCI	IF	3 years	Ketones Cognition	↑Ketogenesis ↑cognition	(89)

Similarly, IF has been shown to enhance phagocytic activity and reduce lipid droplet accumulation in microglia, thereby ameliorating amyloid deposition in APP/PS1-AD mice (double transgenic mice expressing a chimeric mouse/human amyloid precursor protein and a mutant human presenilin 1) (41). These findings suggest fasting contributes not only through systemic metabolic improvements but also by directly modulating AD pathology (54). Mechanistically, fasting suppresses mTORC1 signaling (54), promotes autophagy (45, 79), and upregulates BDNF (54, 80). In one study, male 3XTg-AD mice subjected to ADF for 20 weeks showed significantly increased systemic βOHB, enhanced brain ketone metabolism, restored ATP production, and reduced ROS levels, thereby counteracting mitochondrial bioenergetic deficits commonly observed in AD (10). Together, these adaptations contribute to neuroprotection against AD.

Behavioral studies provide further support. Six-month-old APP/PS1 mice exposed to IF for 1 month showed significant improvements in cognitive performance (41). Likewise,12-month-old 3xTG mice maintained on CR with a fasting period outperformed ad-libitum (AL)-fed controls in the novel object recognition (NOR) task, a measure of recognition memory (54). Yu et al. (10) reported that IF also improved anxiety-like behavior and hippocampal plasticity, while reducing amyloid oligomers. Notably, these benefits occurred without adverse effects on muscle functions or exercise tolerance (10). Furthermore, when male 3xTg-AD rats underwent a prolonged early-life fast and were tested at 17 months, they exhibited improved spatial memory in the memory test; however, IF failed to reduce both hippocampal tau and Aβ levels at this advanced age (59). This suggests that the timing of IF may be critical, and optimal duration and onset remain unresolved even in preclinical models.

Additionally, studies show that 3 months of IF promotes neurogenesis (81) and neuronal differentiation and maturation (82), likely mediated by enhanced BDNF signaling. Specifically in the hippocampal dentate gyrus in 3xTg-AD mice, IF upregulates glycogen synthase kinase 3 (GSK-3) signaling, which is essential for brain development. Interestingly, fasting also reduced insulin signaling while improving adenosine monophosphate-activated protein kinase (AMPK) signaling (82). Majd et al. (53) proposed that one aspect of AD metabolic dysfunction involves hyperactivation of the AMPK-mTOR metabolic axis, leading to abnormal neuronal energy metabolism and AD pathology. They conclude that both AMPK and mTOR are highly upregulated in AD brains, suggesting that the inhibitory link between them is disrupted (53). Whether fasting-induced AMPK activation is a direct regulatory mechanism or a secondary effect of AMPK-mTOR dysregulation in AD remains unknown. Nevertheless, most studies consistently report that IF suppresses mTOR signaling. In peripheral tissues, AMPK activation is essential for metabolic adaptation during fasting (76). These findings underscore the importance of examining the mTOR-AMPK metabolic axis as a regulator of both systemic and brain energy during fasting.

### 4.4 Evidence of IF’s beneficial effects in AD and MCI from clinical trials

The IF has been shown to improve metabolic health in patients with metabolic diseases (Table 2) (2, 83). Human evidence supports improvements in metabolic functions. For example, obese, overweight, and prediabetic individuals subjected to ADF significantly reduced body weight and body mass index compared to controls, whereas 16/8 and 20/4 (wherein individuals fast for 20 h with a 4 h window for caloric intake) time-restricted feeding regimens did not produce significant changes (84). A study by Obermayer et al. found that IF is a safe dietary approach for patients with type 2 diabetes receiving insulin therapy. They have improved glycemic control and reduced daily insulin dose (83). Similarly, patients with metabolic syndrome who followed a two-day modified IF protocol for 8 weeks significantly reduced fat mass, oxidative stress, and inflammatory cytokine levels, and improved vasodilation (85). Individuals introduced to IF also showed favorable alterations in gut microbiota, all of which are linked to reduced cardiometabolic risk (2). In healthy adults, an 18-h daily fast for 1 month reduced body weight, body mass index (BMI), and fat-free mass in both sexes, while lowering resting energy expenditure (REE). Notably, the reduction in REE was greater in females than in males (8.1% vs. 4.6%) (86). This underscores the importance of measuring sex as a variable in IF studies.

While rodent models strongly support IF as a strategy to reduce AD pathology and improve behavioral outcomes, the human evidence is limited but is steadily growing. Several clinical trials suggest metabolic and behavioral alterations with fasting. For example, cognitively intact older adults with insulin resistance who undergo 5:2 IF for 8 weeks showed reduced neuronal insulin resistance and improved memory functions (4). However, despite weight loss and memory improvement, the cerebrospinal biomarkers of AD pathology (amyloid beta, tau, and neurogranin) were not significantly altered (4). A study by Bartholomew reported that cognitively intact adults engaged in a low-frequency IF regimen (24 h twice a week for 4 weeks, then once weekly for 22 weeks) reduced metabolic syndrome score, but showed no improvements in cognitive markers (87). Faris et al. (88) investigated the effects of Ramadan fasting, on circulating inflammatory and immune markers in 50 healthy adults. During the month of Ramadan, healthy adult Muslims observe a daily fast that involves complete abstinence from food and water, from dawn (suhoor) until sunset (iftar). Blood samples were collected before Ramadan and in the third week of fasting, showing significant reductions in proinflammatory cytokines, including IL-1β (78%), IL-6 (57%), and TNF-*α* (71%), as well as decreases in body mass, BMI, and body fat percentage (88). These findings suggest that IF attenuates systemic inflammation, providing a potential mechanism by which fasting may lower risk for chronic neuroinflammation. A 36-month longitudinal study involving older adults (above 60 years old) with MCI found that IF significantly improved cognitive outcomes compared to irregular or no fasting groups, possibly through ketogenesis. Individuals practicing regular IF found that there were overall improvements in weight (3–4%), reduction in body weight and BMI (89).

### 4.5 Differential neuroprotective effects of intermittent fasting across sexes

Emerging evidence suggests that sex specific adaptations to IF in skeletal muscles may contribute to improved neuroprotective outcomes in AD. Preclinical studies demonstrate that, in response to a 24-h fast, females exhibit significant upregulation of BDNF in skeletal muscle (72). This finding highlights a critical role for BDNF in metabolic adaptation to fasting in females, as its deficiency is associated with impaired muscle strength and function (72). Notably, BDNF also promotes fatty acid oxidation and ketone utilization in the brain, in addition to regulating synaptic plasticity and neurogenesis (20). Our pilot study data further support these sex-dependent differences. In females, fasting did not significantly alter muscle or fat mass, whereas males showed a reduction in both lean and fat mass compared to ad libitum-fed males. These findings align with clinical observation from fasting studies in humans, where both sexes specific metabolic responses showed a reduction in body weight and fat-free mass; however, resting energy expenditure (REE) declines more significantly in females (8.1%) compared to males (4.6%) (87). This greater reduction in REE suggests that females may adapt to a more energy-conservative metabolic phenotype during energy restriction, potentially preserving tissue mass while optimizing fuel utilization. Importantly, these metabolic adaptations in females may impact the cognitive benefits of fasting in AD mice. In 3xtg-AD mice, combined CR and fasting reduced amyloid pathology in both sexes; however, only females exhibited significant improvements in cognitive functions (54). In contrast, our recent pilot studies examining the mTOR pathway in males revealed that IF significantly downregulated cortical mTOR expression and improved cognitive performance compared to ad libitum-fed males; this effect was not observed in females (90). These findings suggest that males and females may derive cognitive and neuroprotective benefits from IF through distinct mechanistic pathways.

### 4.6 Safety in older adults with AD

Prolonged dietary interventions in older adults with AD raise important safety concerns, as aging is associated with a decline in skeletal muscle mass and function, and fasting may exacerbate this sarcopenic condition. Evidence from preclinical studies indicates that while fasting reduces fat mass, it may also lead to concomitant loss of lean mass when on a normal diet. These findings highlight the potential risk of accelerating sarcopenia in the older population. To mitigate these risks, IF in older adults should be modified to ensure sufficient protein intake and support muscle protein synthesis. Pharmaceutical interventions such as liraglutide, a glucagon-like peptide-1 receptor agonist, have demonstrated neuroprotective potential in the AD population (91). Clinical studies indicate that liraglutide is generally safe for 52 weeks, but its use is associated with gastrointestinal side effects and clinically significant weight loss. In treated individuals, approximately 8.9% experienced ≥10% weight loss, while up to 39.2% experienced ≥5% weight loss (91). Although some degree of fat mass reduction may be beneficial, excessive or unmonitored weight loss in older adults can increase the risk of sarcopenia. Notable, a study observed that 24 weeks of liraglutide treatment resulted in fat mass reduction while maintaining adaptations to prevent sarcopenia (preserved muscular tropism) (92). However, these adaptations vary among individuals and may depend on baseline nutritional status, physical activity, and diet. Taken together, both dietary interventions (e.g., IF) and GLP-1–based therapies emphasize the need for careful monitoring in older adults with AD. Long-term interventions should include routine assessment of body composition, muscle mass, and strength to minimize the risk of sarcopenic progression.

## 5 Conclusion and future directions

The IF may enhance metabolic resilience in the brain by modulating nutrient-sensing pathways, such as mTOR, which are tightly linked to brain energy metabolism. Hyperactivation of the mTOR pathway has been observed in post-mortem AD brains, and IF has been shown to downregulate it. This suggests that targeting the mTOR pathway may be a promising strategy to improve brain health in AD with IF intervention.

In addition, IF promotes autophagy induction by suppressing mTOR, thereby facilitating the clearance of amyloid beta and tau, key pathological features of AD. This enhancement of autophagy also intersects with dampened innate immune activation, contributing to reduced neuroinflammation. Preclinical studies also demonstrate that IF decreases glial activation (via LCN) and proinflammatory markers, thereby reducing neuroinflammatory signaling in the brain.

Beyond the brain, peripheral tissues such as adipose tissue and skeletal muscle play a critical role in mediating the brain’s benefits from IF. IF has been shown to reduce inflammatory adipokines in both adipose and brain tissues. Furthermore, IF has been shown to preserve skeletal muscle mass and enhance myokine (BDNF) release. This coordinated crosstalk may contribute to systemic improvements in metabolism and supporting brain health in AD.

Despite strong preclinical evidence, gaps remain in translating these mechanisms to AD progression and therapeutic strategies. Future work should examine IF intervention across different brain stages (young, middle-aged vs. aged), explore sex-specific responses, and investigate mechanistic pathways in both the brain and peripheral tissues. Elucidating how IF interacts with age, sex, and genetic risk will strengthen the mechanistic rationale for clinical trials and identify personalized interventions that prevent AD progression.
